# Reduction of N-Acetylglucosaminyltransferase-I Activity Promotes Neuroblastoma Invasiveness and EGF-Stimulated Proliferation In Vitro

**DOI:** 10.3390/ijtm4030035

**Published:** 2024-08-06

**Authors:** Adam P. Burch, M. Kristen Hall, Debra Wease, Ruth A. Schwalbe

**Affiliations:** Department of Biochemistry and Molecular Biology, Brody School of Medicine, East Carolina University Greenville, 600 Moye Boulevard, Greenville, NC 27834, USA

**Keywords:** oligomannose, N-glycans, cell invasion, cell proliferation, neuroblastoma, N-acetylglucosaminyltransferase-I (GnT-I), EGFR, 3D cell culture, MGAT1

## Abstract

Aberrant N-glycosylation has been associated with progression of the pediatric cancer neuroblastoma (NB) but remains understudied. Here we investigated oligomannose N-glycans in NB by genetic editing of *MGAT1* in a human NB cell line, BE(2)-C, called BE(2)-C(MGAT1^−/−^). Lectin binding studies confirmed that BE(2)-C(MGAT1^−/−^) had decreased complex and increased oligomannose N-glycans. The relevance of 2D and 3D cell cultures was demonstrated for cell morphology, cell proliferation, and cell invasion, thereby highlighting the necessity for 3D cell culture in investigating cancerous properties. Western blotting revealed that oligomannosylated EGFR had increased autophosphorylation. Proliferation was decreased in BE(2)-C(MGAT1^−/−^) using 2D and 3D cultures, but both cell lines had similar proliferation rates using 3D cultures without serum. Upon EGF treatment, BE(2)-C(MGAT1^−/−^), but not BE(2)-C, showed increased proliferation, and furthermore, the mutant proliferated much faster than BE(2)-C under 3D conditions. Cell spheroid invasiveness was greatly increased in BE(2)-C(MGAT1^−/−^) compared with BE(2)-C. Moreover, invasiveness was reduced in both cell lines with either EGF or RhoA activator treatment, regardless of the N-glycan population. Thus, this study further extends our earlier findings that oligomannose N-glycans enhance NB cell invasiveness, and that EGF stimulation of oligomannosylated EGFR greatly enhances cell proliferation rates, underlining the role of oligomannose N-glycans in the promotion of NB.

## 1. Introduction

The pediatric cancer, neuroblastoma (NB), is a devastating disease accounting for 15% of pediatric cancer-related deaths [1,2]. Despite recent advances in the treatment of NB, high-risk NB patients have a less than 50% 5-year survival rate due to failure to identify the tumor at an early stage, drug resistance, and tumor relapse [1,3–5]. Drug resistance and cancer relapse are attributed to the high genetic heterogeneity of NB tumors [1,4]. About half of NB patients suffer from drug side effects ranging from high-frequency hearing loss to developmental defects [5,6]. To improve the quality of life for NB patients and to avoid side effects from treatment, new therapeutic and diagnostic targets are needed.

**Figure 1.**
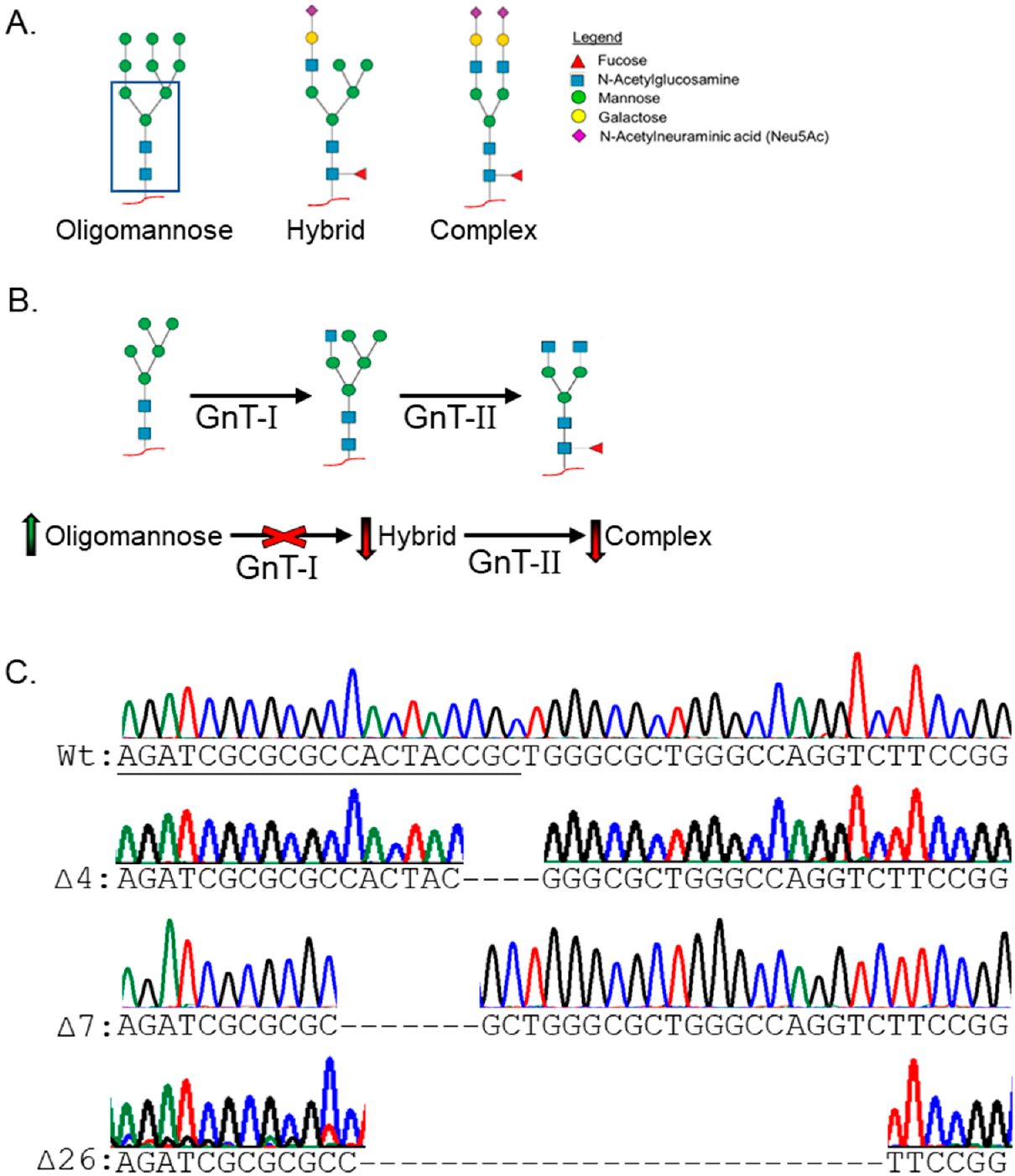
Knockout of *MGAT1* blocks hybrid and complex N-glycan synthesis. (**A**) There are three general types of N-glycans: oligomannose, hybrid, and complex. N-glycans attach to N-glycosylation sites (Asn-X-Ser/Thr) in glycoproteins (red lines). The conserved pentasaccharide is enclosed with a blue line. Oligomannose N-glycans have 0–6 mannose (Man) residues attached to the pentasaccharide. Hybrid N-glycans have Man residues attached to the Manα1–6 arm (right arm of the pentasaccharide), and at least one N-acetylglucosamine (GlcNAc) attached to the Manα1–3 arm (left arm of the pentasaccharide). Replacement of the Man residues with a GlcNAc residue on the right arm of hybrid N-glycans produces complex N-glycans. Complex N-glycans can have up to five branches initiated by GlcNAc and elongated with various monosaccharides. A fucose (Fuc) residue attached to the core is a common modification of hybrid and complex types of N-glycans. An N-acetylneuraminic acid (Neu5Ac) residue commonly caps hybrid and complex N-glycans. In some cases, a chain of Neu5Ac residues is attached to N-glycans, referred to as polysialylated N-glycans. (**B**) N-glycan processing, where oligomannose N-glycans are converted to hybrid N-glycans via GnT-I and then from hybrid to complex N-glycans via GnT-II. Green and red arrows show that the loss of GnT-I (*MGAT1*) results in the accumulation of oligomannose and reduced levels of hybrid and complex N-glycans, respectively. This is a simplified cartoon of the N-glycosylation pathway. (**C**) DNA sequences and chromatograms of the *MGAT1* gene showing the wild type (Wt) sequence in the top panel, and the three frameshift mutations in the lower panels. The frameshift mutations result from deletions of 4, 7, and 27 nucleotides within the *MGAT1* coding sequence.

One promising target is the N-glycosylation pathway as hindrances to this pathway reduce NB proliferation and spreading. N-glycosylation is a co-/post-translational protein modification that begins when the nascent protein enters the lumen of endoplasmic reticulum (ER) and then continues and ends in the lumen of the Golgi apparatus [7]. There are three general types of N-glycans, all of which share a common pentasaccharide core: oligomannose (only mannose (Man) residues attached to the pentasaccharide), hybrid (Man residues trimmed, with an N-acetylglucosamine (GlcNAc) added to the Manα1,3 arm of the pentasaccharide), and complex (Man residues removed and with the addition of a GlcNAc residue to the Manα1,6 arm of the pentasaccharide) (Figure 1A). In the Golgi apparatus, oligomannose N-glycans are converted to hybrid N-glycans, and then hybrid N-glycans can be converted to complex N-glycans by N-acetylglucosaminyltransferase-I (GnT-I) and GnT-II, respectively (Figure 1B, upper panel). After initiation of the various branches by the GnTs, extensions of the branch points are catalyzed by a myriad of glycosyltransferases that link various monosaccharides to create up to five antennae [7]. Ultimately, N-glycan processing is an essential protein modification that is capable of extraordinary structural diversity and tailored to the needs of the glycoprotein, cell, and organism. However, N-glycan processing of proteins is not without consequence, as aberrant N-glycosylation is a common attribute of many physiological disease states, including cancer, e.g., NB [2]. Recently, we have shown that human and rat NB cells expressing more oligomannose N-glycans are more invasive, implicating oligomannose N-glycans as indicators of aggressive NB [8,9]. In fact, oligomannose N-glycans have been associated with the progression of breast, ovarian, liver, and prostate cancer, but they remain understudied [8,10–13]. Therefore, this current study was undertaken to further substantiate the role of oligomannose N-glycans in promoting NB development and progression by engineering NB cells enriched with oligomannosylated proteins, including the oncogenic receptor tyrosine kinase, epidermal growth factor receptor (EGFR).

Several reports have shown a link between NB progression and the restriction of N-glycosylation processing of proteins, including N-glycosylation site occupancy and the processing of N-glycans attached to glycoproteins. For example, reduced occupancy of the N-glycosylation sites of anaplastic lymphoma kinase (ALK) [14] and the reduction of polysialylated N-glycans associated with neural cell adhesion molecule (NCAM) [15] decreased cell proliferation. Further reduced occupancy of the N-glycosylation sites of intercellular adhesion molecule 2 (ICAM-2) suppressed metastasis [16]. Xenografts of human NB cell lines expressing NCAM and N-glycans with polysialic acid into SCID mice showed a promotion of dissemination [17]. A more recent study examined N-glycan profiles in sera from NB patients which revealed differences between NB patients and non-malignant controls [18]. Thus, it is of utmost importance to identify the contribution of specific N-glycan structures on tumor growth and invasion for designing effective NB treatments.

A therapeutic target in many cancers, EGFR (also known as HER1 or ErbB1), is a receptor protein implicated in cell proliferation and invasiveness [19,20]. Upon ligand binding, EGFR monomers dimerize and autophosphorylation occurs, which then initiate downstream signaling before being internalized and fated for degradation or to be returned to the cell surface [21]. It is worth noting that EGFR monomers are capable of dimerizing with a wide range of proteins to initiate downstream signaling; these include, but are not limited to, other EGFR monomers or members of the HER family (e.g., HER2, HER3, or HER4) [21]. Although EGFR favors the formation of homodimers, it is frequently observed in heterodimerization with HER2, which leads to drug resistance and cancer progression [21,22]. By understanding the N-glycosylation status of EGFR and how N-glycosylation of EGFR varies, this may provide a unique therapeutic window to improve EGFR-directed therapies.

EGFR is a heavily N-glycosylated protein bearing mostly complex type N-glycans [23]. Changes in the N-glycan processing of EGFR can promote or diminish EGFR signaling, with the complete inhibition of N-glycosylation greatly reducing EGFR signaling [23–27]. EGFR has been observed in NB tissues and is highly expressed in human NB cell lines [28,29]. However, studies are lacking, and the role of EGFR in the nervous system and NB tumorigenesis are likely underestimated [30]. EGFR has up to thirteen N-glycans, and ten of them are complex type N-glycans [23]. Further, the N-glycans of EGFR have been shown to contribute to both the function and regulation of EGFR [25,26,31]. Changes to the N-glycan processing of EGFR have been shown to both promote or diminish EGFR activation and downstream signaling; however, most studies used chemical inhibition of the N-glycosylation process, which renders the receptors non-functional, or altered the expression of glycosyltransferases, which can increase or decrease receptor function [23,25–27,31]. Regarding oligomannosylated EGFR, localization of this form of EGFR to the plasma membrane was reduced [24]. Moreover, reduced amounts of complex N-glycans led to decreased inhibition of EGFR signaling by the ganglioside GM3 [31]. Here, we expand upon these studies by using fully N-glycosylated and endogenously expressed EGFR from human BE(2)-C cells and compare its function and downstream influence on the proliferation and invasion to that of EGFR associated with only oligomannose N-glycans from our newly generated BE(2)-C(MGAT1^−/−^) cells.

In the past, our lab has shown the significant role of oligomannose N-glycans in decreasing cell proliferation and increasing invasion in human and rat NB [8,32]. In this study, we extend our prior studies to further investigate the role of oligomannose type N-glycans in promoting NB progression via genetic manipulation of the *MGAT1* gene in human BE(2)-C NB cells, thus creating a cell line expressing predominantly oligomannose type N-glycans, namely BE(2)-C(MGAT1^−/−^). Lectin binding studies corroborated the increased expression of oligomannose concomitant with a decrease in complex type N-glycans relative to the parental cell line. Modifications to the cell morphology, proliferation, and invasion were noted in BE(2)-C(MGAT1^−/−^) when compared with BE(2)-C in various microenvironments, e.g., 2D and 3D cell cultures. Western blotting showed endogenous expression of EGFR and further indicated that oligomannosylated EGFR could undergo autophosphorylation more readily than the EGFR associated with complex N-glycans. Moreover, upon EGF stimulation, there was an increase in cell proliferation in BE(2)-C(MGAT1^−/−^) that was not observed in BE(2)-C. In contrast, cell invasiveness was markedly reduced by either EGF or a RhoA activator, regardless of the N-glycan population. The current study, along with past investigations [8,32], establish that enrichment of the oligomannose type of N-glycans promotes NB development and progression.

## 2. Materials and Methods

### 2.1. Cell Lines and Cell Culture

Human BE(2)-C cells were purchased from American Type Culture Collection (Manassas, VA, USA). CRISPR/Cas9 technology was utilized to edit the gene α-1,3-mannosyl-glycoprotein-2-β-N-acetylglucosaminyltransferase (*MGAT1*) (accession: CR45681) as previously described [8], using sgRNAs designed via the CHOPCHOP web toolbox [33–35]. The sgRNA oligonucleotide used to target *MGAT1* was AGATCGCGCGCCACTACCGC (nucleotides 551–570). In brief, the respective sgRNAs were incorporated into the pSpCas9(BB)-2A-Puro vector (Addgene plasmid ID: 48139) and transfected in BE(2)-C cells to silence *MGAT1* as previously described [36]. Lectin selection (25 μg/mL, Ricinus communis agglutinin (RCA); Vector Laboratories, Inc., Burlingame, CA, USA) of clones was used to identify the functional loss of *MGAT1* and to grow a cell colony with minimal GnT-I activity [32,37]. Genomic fragment sequencing verified that the gene silencing from ten separate cell clones revealed 4-, 7-, and 26-nucleotide deletions, resulting in premature STOP codons at nucleotides 686–688, 686–688, and 630–632, respectively. All cells were maintained at 5% CO_2_ at 37 °C in DMEM containing 10% FBS, 50 U/mL penicillin, and 50 μg/mL streptomycin. BE(2)-C(-*MGAT1*) cells were rescued via transient transfection with a pCDNA3.1 vector containing the mouse *MGAT1* cDNA. The *Mgat1* CDS was a kind gift from Dr. Pamela Stanly, College of Albert Einstein [38], and used for cloning into the PCDNA3.1 vector [8].

### 2.2. 3D Cell Spheroid Formation

3D cell spheroids were grown in spherical 5D plates (Kugelmeiers, Erlenbach, Sweden) as previously described [8]. In short, 0.5 mL of DMEM media was added to wells of the dish and briefly spun (3 min/500× *g*) to remove air bubbles. Cells (7.5 × 10^4^ cell/mL per well) were added and allowed to form spheroids overnight (37 °C, 5% CO_2_) in DMEM containing 50 U/mL penicillin, 50 μg/mL streptomycin, and with or without 10% FBS, depending on the downstream studies.

### 2.3. Whole Cell Lysates

Whole cell lysates were harvested in RIPA buffer (PBS, 1% Triton X-100, 0.5% sodium deoxycholate, 0.1% SDS, protease inhibitor cocktail set III (EMD Biosciences, San Diego, CA, USA)) as previously described [36]. 2D lysates were obtained via the scraping of cells grown to confluence. 3D lysates were harvested from spheroids cultured for seven days. In both cases, cells were sheared using a 20 G needle and then incubated on ice for 30 min. Supernatants were retrieved post-centrifugation (15,000× *g*/20 min/4 °C using an Eppendorf F-45–30-11 rotor (Eppendorf, Westbury, NY, USA) and stored at −80 °C until ready for use.

### 2.4. Total Membrane Isolation and Glycosidase Treatment

Total membranes were harvested from cells grown to confluence and resuspended in 10 mM Tris (pH 7.4), 250 mM sucrose, 5 mM EDTA, protease inhibitor cocktail set III (EMD Biosciences, San Diego, CA, USA) buffer as previously reported [36]. Total membrane proteins (105 μg) were subjected to glycosidase treatment using 20 U/L PNGase F or EndoH (New England Biolabs, Ipswich, MA, USA) in the presence of supplied buffers overnight at 37 °C, which was then stopped by the addition of reducing SDS-PAGE sample buffer.

### 2.5. Western and Lectin Blotting

Proteins were separated on 10% SDS-PAGE or Any kD^™^ 12% gels and subsequently transferred to a PVDF membrane (Millipore, Billercia MA, USA), or nitrocellulose membranes (Bio-Rad, Hercules, CA, USA), respectively, as previously described [36]. Membranes were blocked with 5% dry milk or EveryBlot Blocking Buffer for fluorescent Westerns (Bio-Rad, Hercules, CA, USA) followed by probing with biotin-conjugated E-PHA, L-PHA, or GNL lectins (Vector Laboratories, Burlingame, CA, USA), or antibodies (EGFR or pEGFR; Cell Signalling Technology, Danvers, MA, USA) overnight. Following washes (1X PBS + 1% Tween20; lectin blots) or (TBS + Tween20; Western blots), the membranes were incubated with streptavidin or secondary antibodies (anti-rabbit IgG, starBright Blue 700; Bio-Rad, Hercules, CA, USA). Blots were developed with NBT/BCIP (lectins). Fluorescent blots were imaged using the Bio-Rad ChemiDoc MP imaging system (Bio-Rad, Hercules, CA, USA). Multiplexed Western blots contained the band of interest multiplexed with β-actin as the loading control. Immunobands were quantified using Bio-Rad’s Image Lab software (Version 3.1). Quantification of the Westerns is shown as the ratio of $\frac{\mathrm{Band}\;\mathrm{of}\;\mathrm{Intrest}}{\beta -\mathrm{actin}}$.

### 2.6. Cell Dissociation Assay

Dissociation of the cells was assayed as previously described [8]. Briefly, confluent cells on CellBind Culture dishes (Corning, NY, USA) were washed (2X) with DMEM and then detached via scraping. Cells were then dissociated via pipetting using a 1 mL pipette tip. Cell cluster mages were obtained using an A IX71 Olympus microscope (Olympus, Tokyo, Japan) with a 20× objective, and the area of cell clusters <10 cells was measured using Image J software, version 1.51.

### 2.7. 2D and 3D BrdU Proliferation

5-bromo-2-deoxyuridine (BrdU) (Millipore, Billerica, MA) internalization was used to assay cell proliferation. The manufacturer’s protocol was followed as previously described [8]. In short, 2 × 10^4^ dispersed cells or formed spheroids were pipetted into individual wells of a 96-well dish (cell culture treated for 2D assay; suspension culture plate for 3D assay) and incubated in the presence of BrdU reagent for 22 h under standard culturing conditions. Following the overnight incubation, cells were fixed for 30 min at room temperature, incubated with an anti-BrdU monoclonal antibody, then incubated with a secondary goat-α-mouse IgG peroxidase conjugate, and the absorbance was measured at 450 nm using a Multiskan FC plate reader (Fisher Scientific, Atlanta, GA, USA). The resulting absorbances were averaged, and the resulting values were calculated as follows: $averageABS_{+BrdU}-average\;ABS_{-BrdU}=\;Relative\;Rate\;of\;Proliferation$.

### 2.8. Anchorage Independent Growth

The soft agar assay was used to assay anchorage-independent growth as indicated in previous reports [8]. In short, a 1:1 mixture of 1% noble agar and 2X DMEM was placed into the bottom of a 35 mm dish and allowed to solidify. Then, a solution of 8.5 × 10^3^ cell/mL (in DMEM) was mixed 1:1 with melted (but cooled) 0.6% noble agar and allowed to solidify. An amount of 0.5 mL DMEM was added to the top to avoid the agar from drying, and the plate was allowed to incubate under standard cell culture conditions for 14 days. Following the 14-day incubation, images were captured using a IX71 Olympus microscope (20×), and the area of the cell growth was measured using Image J software, version 1.51.

### 2.9. Morphology

Cells were plated at low density and incubated for 20 h before being imaged on an Olympus IX71 microscope with a 20× objective. Cells were then classified as round cells, round cells with projections, or flat cells, and the percentage of each cell morphological type per field was scored.

### 2.10. 2D Migration and Invasion

BD Falcon cell chambers (BD Biosciences, San Jose, CA, USA) were utilized to examine cell migration as previously noted [8]. Cells were plated at 2.5 × 10^4^ cells in serum free DMEM and pipetted into a transwell insert without (migration) or with Matrigel (invasion) in a 24-well culture dish. Sera-free DMEM (migration) or DMEM containing 10% FBS (migration) was placed in the lower chambers of the dish. Cells were allowed to migrate (4 h) or invade (22 h) at 37 °C. Membranes, without and with Matrigel, were fixed with 100% methanol and stained with 1% Toluidine blue, followed by excision of the membrane from the insert, which was then affixed to a microscope slide for cell counting and imaging. Cells from each membrane were counted using a Nikon TMS microscope (Nikon, Tokyo, Japan) to determine the number of migratory or invasive cells per membrane. Images were obtained using an Olympus IX73 microscope (20× objective).

### 2.11. 3D Spheroid Invasion

One-day-formed cell spheroids were harvested, allowed to settle, collected, pipetted into pre-cooled Matrigel (Corning, Corning, NY, USA), and gently mixed to avoid disruption of spheroids as we previously described [8]. Next, 40 μL of the spheroid–Matrigel solution was pipetted into a 24-well dish and incubated at (37 °C) for 30 min to allow the Matrigel to solidify, followed by the addition of either 1 mL of complete DMEM or, for EGF studies, serum-free DMEM without or with EGF. Spheroids were allowed to invade for 22 h. Spheroid invasion was imaged using an IX71 Olympus microscope (10× objective). The invasion area and cell spheroid area were measured with Image J software, version 1.54d, and the relative invasiveness of the cells was calculated as follows: InvasionArea=InvasiveArea/SpheriodArea

### 2.12. EGF Treatment

Cells utilized for EGF treatment to assay cell proliferation and invasion were initially either grown to confluence (2D) or allowed to form spheroids overnight (3D). In all cases, cells were then serum-starved for 22–24 h. Consequently, cells were treated with or without 20 ng/mL recombinant human EGF (ThermoFisher Scientific, Waltham, MA, USA), and proliferation and invasion assays were carried out as stated above. Cells used for dose response studies were serum-starved for 24 h, treated without or with various amounts of EGF (0, 20, and 100 ng/mL) for 2 min at 37 °C, and then lysates were harvested in RIPA buffer (see above) plus 2× Protease/Phosphatase inhibitor (Thermofisher Scientific, Waltham, MA, USA).

### 2.13. RhoA Activation to Examine Invasion

One-day-formed cell spheroids were serum-starved for 24 h, collected, and allowed to invade for 24 h as described above. Spheroid invasion occurred without or with Rho Activator II (1 μg/mL) (Cytoskeleton, Inc., Denver, CO, USA). Images were obtained using an IX71 Olympus microscope (10× objective).

### 2.14. Data Analysis

All data are presented as the mean ± standard error of the mean (SEM). Where indicated, “*n*” denotes the number of any given observation. Unpaired Student’s *t*-test were used to compare two groups, while one-way ANOVAs with the post hoc Holm–Bonferroni means comparison test were used to compare more than two groups. In instances in which there were two levels of comparison, a two-way ANOVA was used with a post-hoc Holm–Bonferroni means comparison test.

## 3. Results

### 3.1. Manipulation of the N-glycan Processing Pathway to Yield Oligomannosylated EGFR

**Figure 2.**
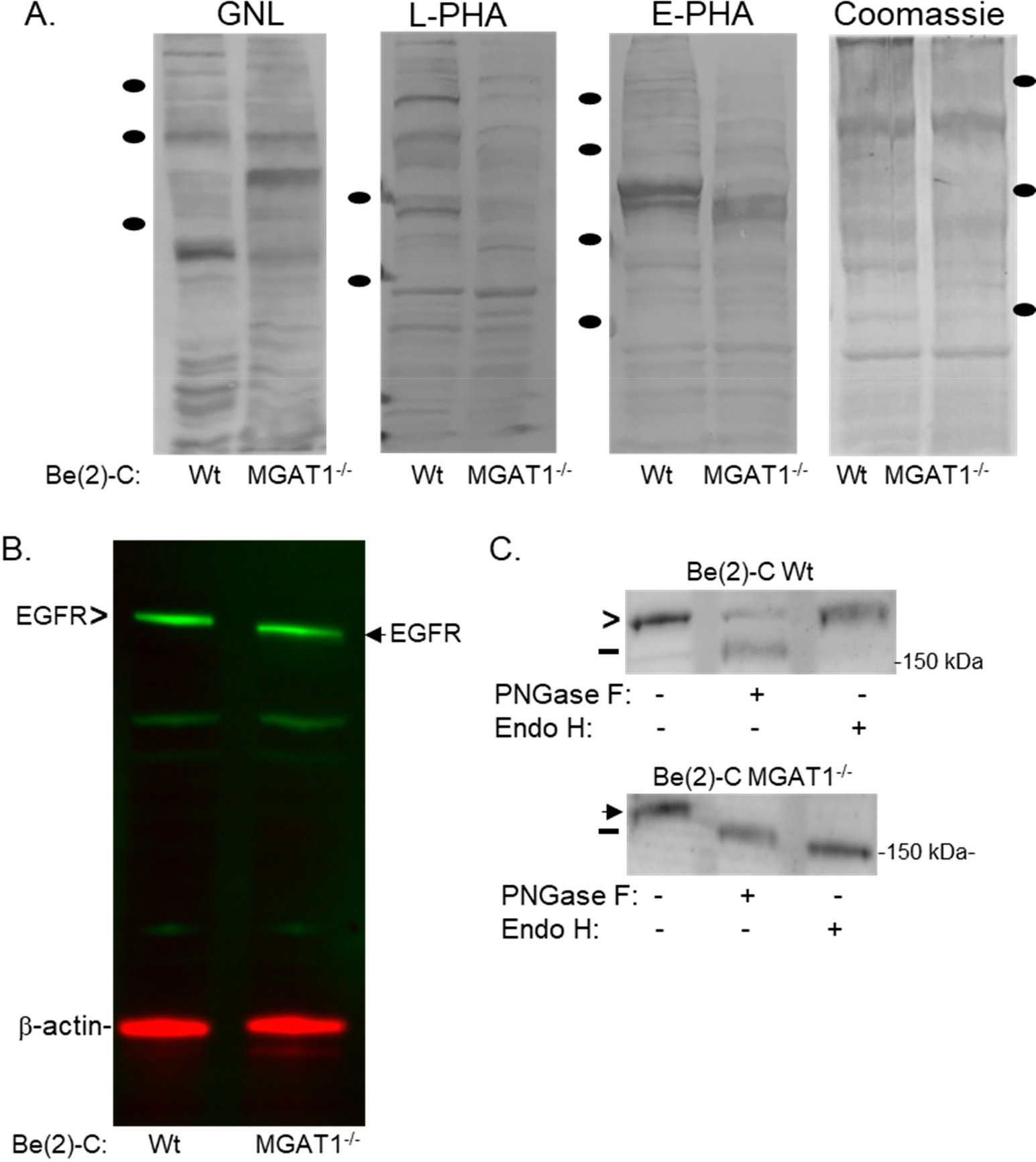
Altered lectin binding and oligomannosylated EGFR from BE(2)-C(MGAT1^*−*/*−*^) cells. (**A**) GNL, L-PHA, and E-PHA lectin blots, along with Coomassie blue-stained gels, of whole cell lysates from BE(2)-C and BE(2)-C(MGAT1^−/−^) cells. Black-filled ovals represent molecular weights markers of 150, 100, and 75 kDa for the lectin blots and the Coomassie blue gel; only 100 and 75 kDa markers are shown for the L-PHA blot. (**B**) A multiplexed EGFR/β-actin Western blot of both BE(2)-C and BE(2)-C(MGAT1^−/−^) whole cell lysates. (**C**) EGFR Western blot of total membranes from BE(2)-C and BE(2)-C(MGAT1^−/−^) (+) digested or (−) not digested with PNGase F or Endo H. The chevron signifies EGFR that is associated with complex N-glycans while the black arrowhead denotes oligomannosylated EGFR, and the black line represents EGFR with N-glycans removed.

Using CRISPR/Cas9 knockout technology, GnT-I expression was reduced in a human clonal NB cell line, BE(2)-C. The BE(2)-C cell line is also referred to as the wild-type (Wt) or parental cell line throughout the text. GnT-I is encoded by *MGAT1* and is an enzyme responsible for the conversion of oligomannose to hybrid N-glycans; this step is required to produce complex N-glycans. Loss of *MGAT1* prevents cells from producing hybrid and complex N-glycans, and as a result, all glycoproteins, including EGFR, will be associated with oligomannose N-glycans (Figure 1B, lower panel). Using the galactose-binding lectin Ricinus Communis Agglutinin-I (RCA-I) as a selective marker, we were able to establish a BE(2)-C(MGAT1^−/−^) cell line that comprised cells with three different frameshift mutations that included deletions of 4, 7, or 26 nucleotides (Figure 1C). To further validate the functional loss of GnT-I, we used lectin binding studies of whole cell lysates from parental and *MGAT1* knockout cells. GNL, L-PHA, and E-PHA bind with high affinity to oligomannose N-glycans, tri- and tetra-antennary complex N-glycans, and bisecting complex N-glycans, respectively [39]. GNL binding was greatly increased, while L-PHA and E-PHA binding was greatly diminished in the BE(2)-C(MGAT1^−/−^) cells relative to BE(2)-C cells (Figure 2A). Cells with functional loss of GnT-I, and thus expressing high amounts of oligomannose N-glycans, would have increased GNL binding but decreased L-PHA and E-PHA binding, as observed. Altogether, the observed frameshift mutants and altered lectin binding profiles indicate reduced GnT-I activity in the BE(2)-C(MGAT1^−/−^) cell line.

Next, we investigated endogenous expression of EGFR in the BE(2)-C and BE(2)-C(MGAT1^−/−^) cell lines by Western blotting. EGFR was observed in both cell lines at relatively similar levels (Figure 2B). However, the electrophoretic migration of EGFR was noticeably faster in the mutant cell line, indicating that the N-glycans of EGFR are composed of reduced monosaccharides and antenna, thus suggesting fewer processed N-glycans (i.e., oligomannose type N-glycans) than in the parental cell line. To confirm that the N-glycans were of the oligomannose type, we employed Endo H or PNGase F digestion reactions to selectively remove oligomannose N-glycans or all general types of N-glycans, respectively. Total membrane fractions from BE(2)-C and BE(2)-C(MGAT1^−/−^) cells were isolated and either untreated or treated with the various glycosidases. If the EGFR had a faster electrophoretic migration upon treatment than the untreated sample, it would indicate that the glycosylated EGFR was sensitive to a given endoglycosidase. EGFR from BE(2)-C cells was sensitive to PNGase F but resistant to EndoH, while EGFR from BE(2)-C(MGAT1^−/−^) cells were sensitive to both endoglycosidases (Figure 2C). Taken together, these results support that EGFR was mainly associated with complex N-glycans in parental cells and with oligomannose N-glycans in mutant cells; furthermore, the BE(2)-C(MGAT1^−/−^) cell line has minimal GnT-I activity.

### 3.2. Rescue of Altered Cell–Cell Adhesion, Proliferation, and Morphology in the MGAT1 Mutant NB Cell Line

**Figure 3.**
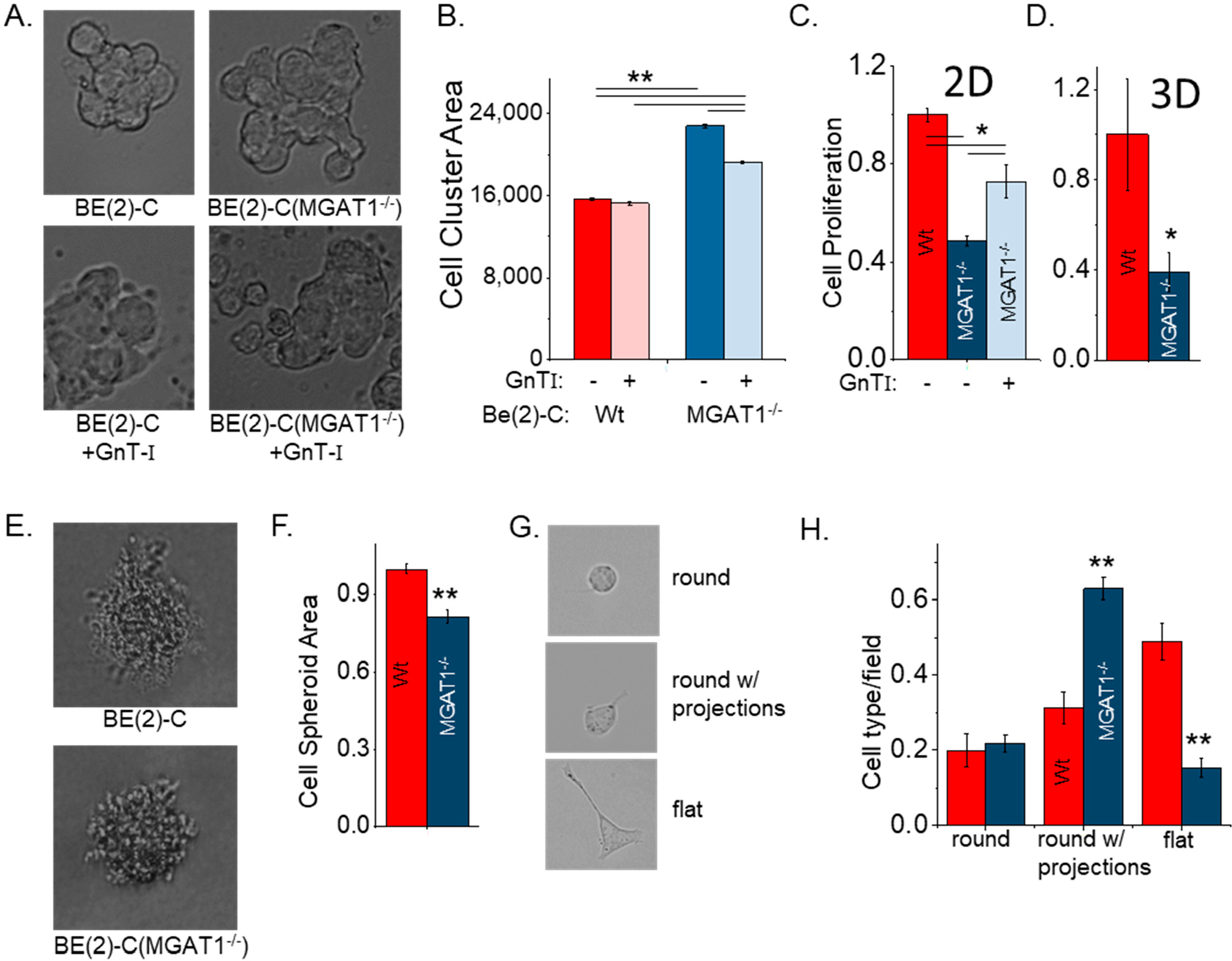
Loss of *MGAT1* impacted NB cell–cell adhesion, proliferation, and morphology. (**A**) Representative DIC images of intact cell clusters and (**B**) the area of cell clusters from BE(2)-C (*n* = 926), BE(2)-C +GnT-I (*n* = 408), BE(2)-C(MGAT1^−/−^) (*n* = 739), and BE(2)-C(+/−*MGAT1*) (*n* = 898) following mechanical dissociation via pipetting. *n* denotes the number of cell clusters. (**C**) BrdU cell proliferation assay under 2D cell culturing conditions for BE(2)-C for Wt with (*n* = 4) and without (*n* = 8) GnT-I. (**D**) 3D cell spheroid BrdU proliferation assay (*n* = 8) normalized to BE(2)-C for Wt and mutant cell lines. (**E**) Representative DIC images of cell colonies from anchorage-independent growth of BE(2)-C and BE(2)-C(MGAT1^−/−^) and (**F**) quantification of the area of clusters from BE(2)-C (*n* = 627) and BE(2)-C(MGAT1^−/−^) (*n* = 172) following 13 days of growth, where *n* is the number of cell clusters. (**G**) Examples of the cell types in BE(2)-C and BE(2)-C(MGAT1^−/−^) cells, as indicated. (**H**) Percentage of cell type per field of Wt and MGAT1 mutant cells (*n* = 59), where n signifies the number of fields examined. In all cases, the data are normalized to BE(2)-C, except in panel (**B**). All quantifications are represented as the mean ± SEM. One-way ANOVA with a post hoc Holm–Bonferroni test was used when comparing more than 2 samples, where * *p*< 0.05 and ** *p*< 0.01. The Student’s t-test was used to compare Wt to MGAT1 mutant in panels (**D**,**F**,**H**), where * *p* < 0.04 and ** *p* < 0.0001.

Previously, we established that rat NB cells solely expressing oligomannose N-glycans had increased cell–cell adhesion, decreased cell proliferation, and overall shorter neurite projections [8]. Here, we investigated whether changes in these cellular properties due to reducing the amount of functional GnT-I could be extended to human NB cells. BE(2)-C(MGAT1^−/−^) cells had increased cell cluster sizes relative to BE(2)-C cells, as viewed in representative images (Figure 3A) and shown by the mean values of the accumulated data (Figure 3B). The cell cluster area was unchanged in the parental cell line when cells were transiently transfected with a vector encoding *Mgat1*. On the other hand, expression of *Mgat1* in the *MGAT1* mutant cells caused a reduction in the cell cluster area, indicating that the increased cell–cell adhesion was due to increased oligomannose N-glycans. Secondly, we demonstrated that cell proliferation via a BrdU proliferation assay was reduced in the *MGAT1* mutant cell line compared with the Wt cell line using 2D (Figure 3C) and 3D (Figure 3D) culturing conditions. Moreover, cell proliferation was increased in *MGAT1* mutant cells heterologously expressing *Mgat1*, indicating that lowered activity of GnT-I was a likely cause of the decreased proliferation. Thirdly, it was observed that Wt cells could marginally better survive without adherence to a tumor or to the extracellular matrix, as the non-adhered cell spheroid area was slightly larger than that of the BE(2)-C(MGAT1^−/−^) cells (Figure 3E,F). Lastly, we compared the cell morphology of the parental cell line to the *MGAT1* mutant cell line. Three different morphologies were observed in both cell lines, including round cells, round cells with projections, and flat cells (Figure 3G). However, the parental cell line had more flat cells, while round cells with neurites made up the majority of the *MGAT1* mutant cell population (Figure 3H). Overall, these studies further support that *MGAT1* expression is reduced in the mutant cell line relative to the parental cell line. Moreover, they reveal that changing the N-glycan population in human NB cells can modify cellular properties and implicate changes in cell signaling events, e.g., in the EGFR signaling pathway.

### 3.3. Oligomannosylated EGFRs Have an Increased Response to EGF Treatment and Sensitize NB Cells to EGF-Stimulated Proliferation

**Figure 4.**
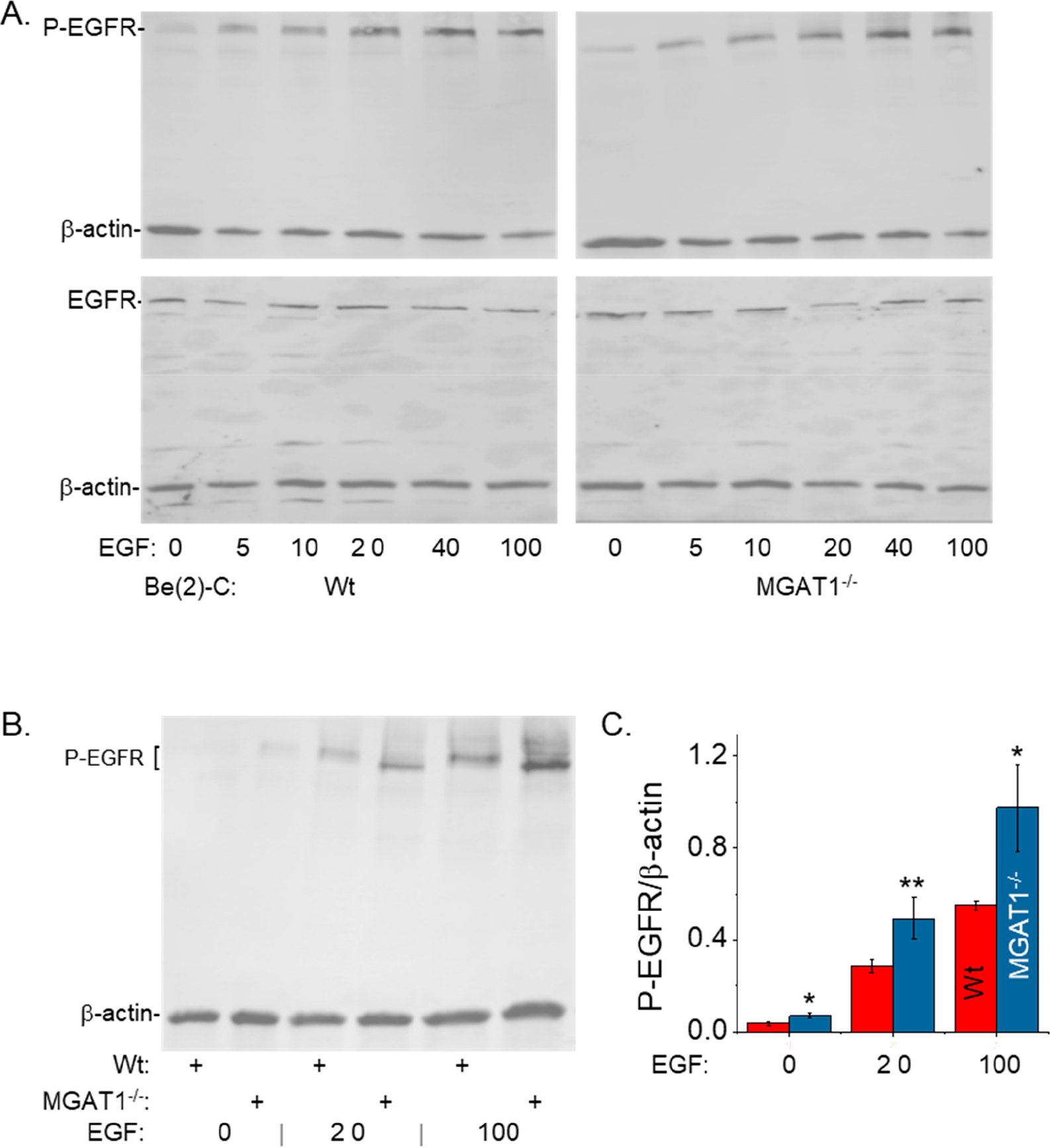
Oligomannosylated EGFR heightened autophosphorylation following EGF stimulation. (**A**) Multiplexed Western blots of pEGFR Y1173/β-actin (**Top**) and pan-EGFR/β-actin (**Bottom**) from BE(2)-C (**Left**) and BE(2)-C(MGAT1^−/−^) (**Right**) following EGF stimulation with EGF, as indicated. (**B**) Multiplexed pEGFR Y1173/β-actin Western blot (*n* ≥ 5) of BE(2)-C and BE(2)-C(MGAT1^−/−^) following 0 ng/mL, 20 ng/mL, and 100 ng/mL EGF stimulation, and (**C**) quantification of the pEGFR immunoband with respect to β-actin. The Student’s *t*-test was used to compare Wt to the MGAT1 mutant, where ** *p* < 0.041 and * *p* < 0.063. All quantifications are represented as the mean ± SEM.

To assess whether the EGFR signaling pathway was altered via oligomannosylated EGFR, we first examined receptor autophosphorylation in response to EGF stimulation at various doses (0–100 ng/mL EGF) by Western blotting for phospho-EGFR (Y1173) (denoted as P-EGFR) and pan-EGFR (signified as EGFR) (Figure 4A). As EGF levels were increased, there was a steady increase in the autophosphorylation of EGFR (P-EGFR) in both the BE(2)-C and BE(2)-C(MGAT1^−/−^) cell lines. However, the expression of EGFR remained relatively constant in both cell lines. Based on the response of EGFR autophosphorylation to EGF, we selected three concentrations of EGF (0, 20, and 100 ng/mL) and measured the level of EGFR autophosphorylation in the cell lines (Figure 4B). Again, P-EGFR immunobands were observed to migrate slightly faster in BE(2)-C(MGAT1^−/−^) cells due to replacement of the complex type N-glycans by oligomannose type N-glycans. Quantification of the intensity of the P-EGFR band with respect to β-actin revealed higher levels of EGFR autophosphorylation in BE(2)-C(MGAT1^−/−^) cells than in BE(2)-C cells in response to 0, 20, and 100 ng/mL EGF (Figure 4C), indicating that oligomannosylated EGFR could more readily undergo autophosphorylation than EGFR associated with complex N-glycans.

**Figure 5.**
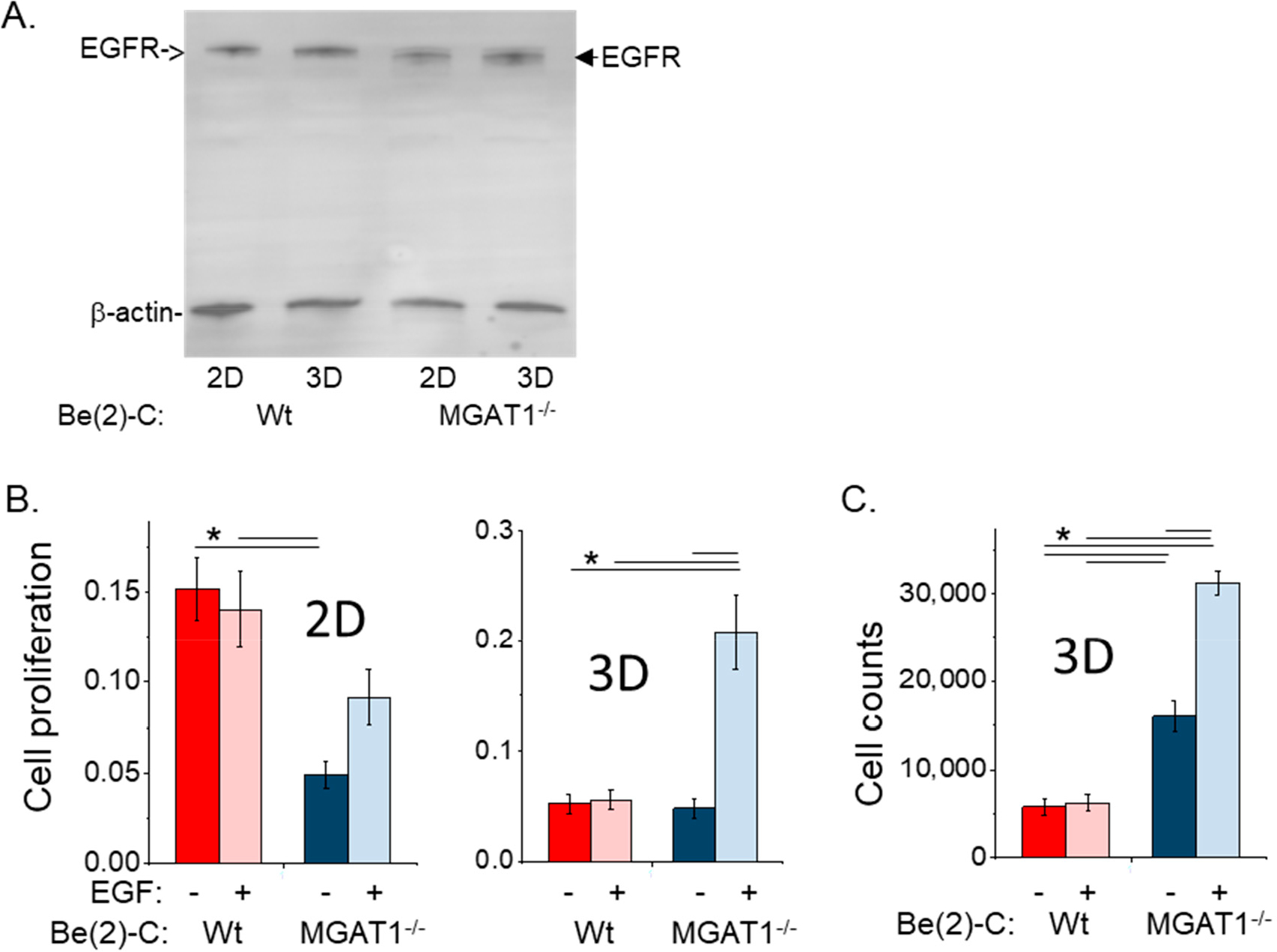
Oligomannosylated EGFR sensitized NB to EGF-stimulated proliferation in 2D and 3D cell cultures. (**A**) Multiplexed Western blot of EGFR/β-actin from either 2D or 3D whole cell lysates of BE(2)-C and BE(2)-C (*MGAT1*^−*/*−^). (**B**) 2D (*n* ≥ 6) and 3D (*n* = 8) BrdU proliferation of BE(2)-C and BE(2)-C (*MGAT1*^−*/*−^) following 20 ng/mL EGF stimulation. (**C**) 3D cell counts (*n* = 6) of BE(2)-C and BE(2)-C(MGAT1^−/−^) following 20 ng/mL EGF stimulation. Two-way ANOVA used with correction; * *p* < 0.05. All quantifications are represented as the mean ± SEM.

Since the phosphorylation of EGFR is crucial to further downstream signaling and essential for EGF-stimulated proliferation [21], cell proliferation of BE(2)-C and BE(2)-C(MGAT1^−/−^) cells in response to EGF treatment was studied using both 2D and 3D cell cultures. Western blotting of EGFR from whole cell lysates of BE(2)-C and BE(2)-C(MGAT1^−/−^) cells showed that the expression of EGFR under 2D cell culturing conditions was quite comparable, and that under 3D cell culturing conditions, the EGFR levels in both cell lines appeared to increase to relatively similar visible levels (Figure 5A). Next, cell proliferation assays with or without 20 ng/mL EGF (Figure 5B) were carried out under 2D culturing conditions. EGF-treated BE(2)-C cells did not show an increase in proliferation relative to untreated BE(2)-C cells; however, EGF-treated BE(2)-C(MGAT1^−/−^) cells showed a significant increase in proliferation relative to its untreated counterpart. Moreover, cell proliferation of BE(2)-C(MGAT1^−/−^) with EGF was close to the sub-level of BE(2)-C with or without EGF. Analysis of cell proliferation under 3D cell culturing conditions for BE(2)-C cells without and with EGF treatment were unaltered, while BE(2)-C(MGAT1^−/−^) cells treated with EGF showed a significant increase in cell proliferation relative to untreated BE(2)-C(MGAT1^−/−^) cells. BE(2)-C(MGAT1^−/−^) cells treated with EGF were 4.2-fold more abundant than those of BE(2)-C(MGAT1^−/−^) without EGF and BE(2)-C, plus or minus EGF. Two-way ANOVA was used to illustrate how cell proliferation (outcome) was changed by the loss of functional GnT-I (mediator) and EGF (moderator). It reported that diminished functional GnT-I reduced cell proliferation, and furthermore, the addition of EGF increased the level of cell proliferation in cells with limited functional GnT-I under 2D cell culturing conditions. On the other hand, cell proliferation was unchanged due to lowered functional GnT-I in 3D cell cultures, but there was a robust increase in proliferation when the GnT-I activity was largely reduced in conjunction with EGF treatment.

To further confirm the remarkable results of the 3D BrdU proliferation study, a similar protocol was conducted, except that cell growth was measured by counting cells instead of measuring cell proliferation by the incorporation of BrdU into DNA (Figure 5C). Cells with reduced functional GnT-I showed an increase of about 2.8-fold in cell growth relative to those cells with an intact GnT-I, and the addition of EGF further increased cell growth under 3D cell culturing conditions, while Wt cells did not respond to EGF treatment. Thus, the cell proliferation and growth assays strongly suggest that oligomannosylated EGFR sensitizes NB cells to EGF-stimulated cell proliferation and growth. The increase in the number of cells in *MGAT1* mutant cells relative to the Wt cells is likely to be because there are viable cells that are not undergoing DNA replication; this is also supported by the smaller increase in cell growth in response to EGF for the mutant cells by cell counting versus BrdU incorporation.

### 3.4. Cell Migration Is Unaltered While Cell Invasiveness Is Suppressed by Nonfunctional GnT-I Using 2D Dispersed Cell Culture

**Figure 6.**
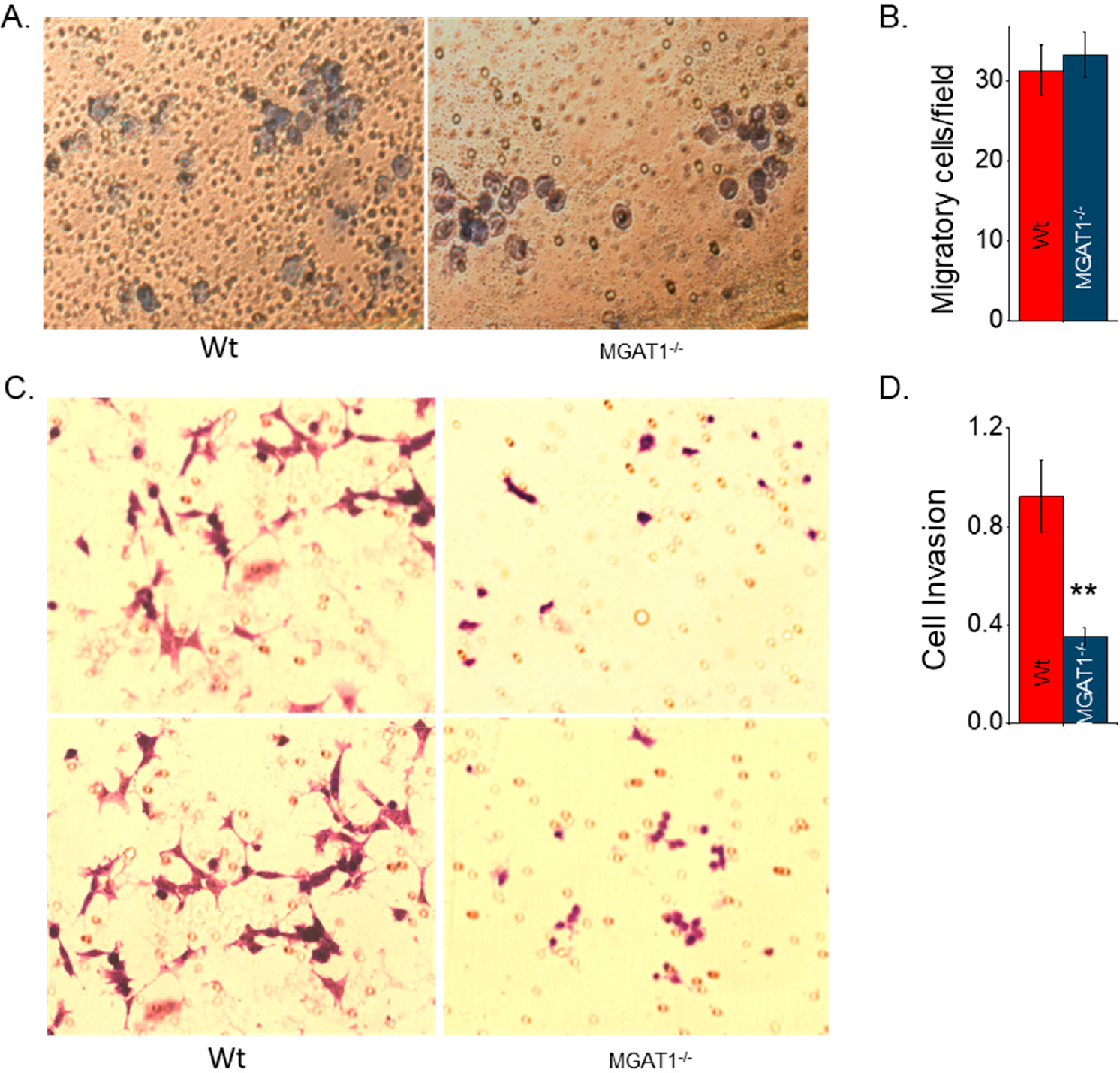
Loss of *MGAT1* decreased 2D invasion and migration. (**A**) Representative images of either BE(2)-C or BE(2)-C(MGAT1^−/−^) cells from the bottom of membrane insert, indicating migration, following 4 h of incubation. (**B**) The average number of migratory cells (*n* = 16) that crossed the membrane insert. *n* represents the number of fields. (**C**) Images of invasive BE(2)-C or BE(2)-C(MGAT1^−/−^) cells following 22 h of incubation and their invasion through a Matrigel invasion chamber. (**D**) Average number (*n* = 5) of BE(2)-C cells and BE(2)-C(MGAT1^−/−^) cells that invaded through the Matrigel chamber. *n* signifies the number of wells. All quantifications are represented as the mean ± SEM. The Student’s *t*-test was used to compare the Wt to the MGAT1 mutant; ** *p* < 0.01.

A low-density aliquot of either parental or *MGAT1* mutant cells was added to a cell chamber to produce a 2D sub-confluent cell culture. After a 4 h incubation, micrographs were acquired that represented the number of migratory cells per field (Figure 6A). The number of migratory cells from each cell line was quantified to demonstrate the reproducibility of the observations (Figure 6B). Next, the cell invasiveness of BE(2)-C and BE(2)-C(MGAT1^−/−^) was examined in 2D sub-confluent cell cultures. BE(2)-C cells could degrade and move through the extracellular matrix boundary much faster than BE(2)-C(MGAT1^−/−^), as shown on representative micrographs (Figure 6C). The average number of invasive cells from each of the cell lines show that BE(2)-C cells were about 2.6-fold more invasive than BE(2)-C(MGAT1^−/−^) cells (Figure 6D). These results show that substitution of complex and hybrid N-glycans with oligomannose N-glycans does not modify the cell migratory rates but greatly decreases the cell invasiveness of human NB cells in 2D sub-confluent cultures.

### 3.5. Oligomannose N-glycans Promote Cell Spheroid Invasiveness, and Both EGF and RhoA Treatment of NB Cell Lines Markedly Suppress Cell Spheroid Invasiveness

**Figure 7.**
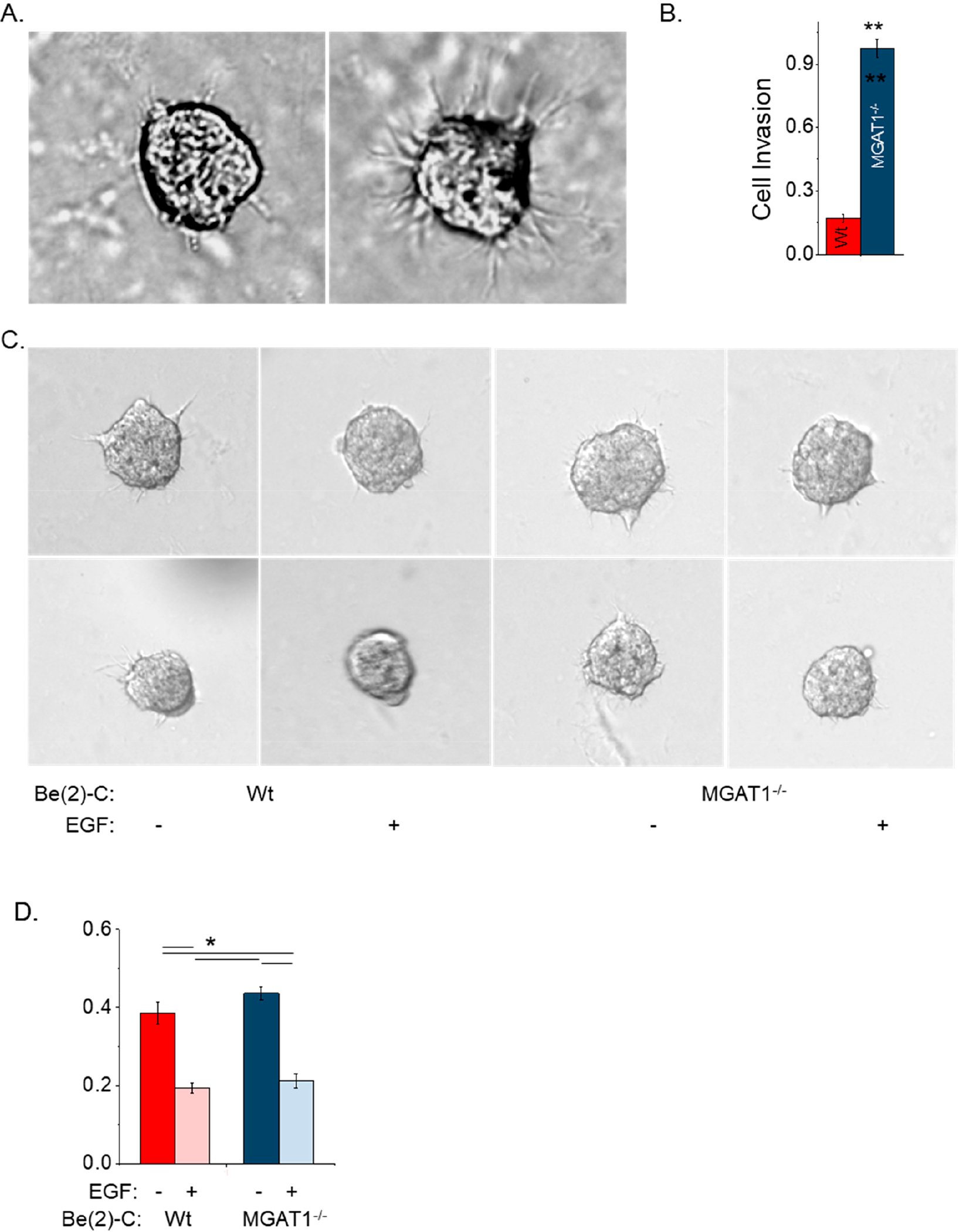
Oligomannose N-glycans increased 3D spheroid invasiveness but EGF treatment impaired 3D invasion in both BE(2)-C and BE(2)-C(MGAT1^−/−^). (**A**) Images of 3D spheroids from BE(2)-C and BE(2)-C(MGAT1^−/−^) following 22 h of invasion. (**B**) Quantification of the invasion area for BE(2)-C (*n* = 75) and BE(2)-C(MGAT1^−/−^) (*n* = 77). The Student’s t-test was used, where ** *p* < 0.0001. (**C**) DIC images of invading spheroids and (**D**) quantification of the invasion area of BE(2)-C without EGF (*n* = 79), BE(2)-C with 20 ng/mL EGF (*n* = 83), BE(2)-C(MGAT1^−/−^) without EGF (*n* = 66), and BE(2)-C(MGAT1^−/−^) with 20 ng/mL EGF (*n* = 35). *n* denotes the number of invading spheroids. Two-way ANOVA was used with correction; * *p* < 0.05. All quantifications are represented as the mean ± SEM.

**Figure 8.**
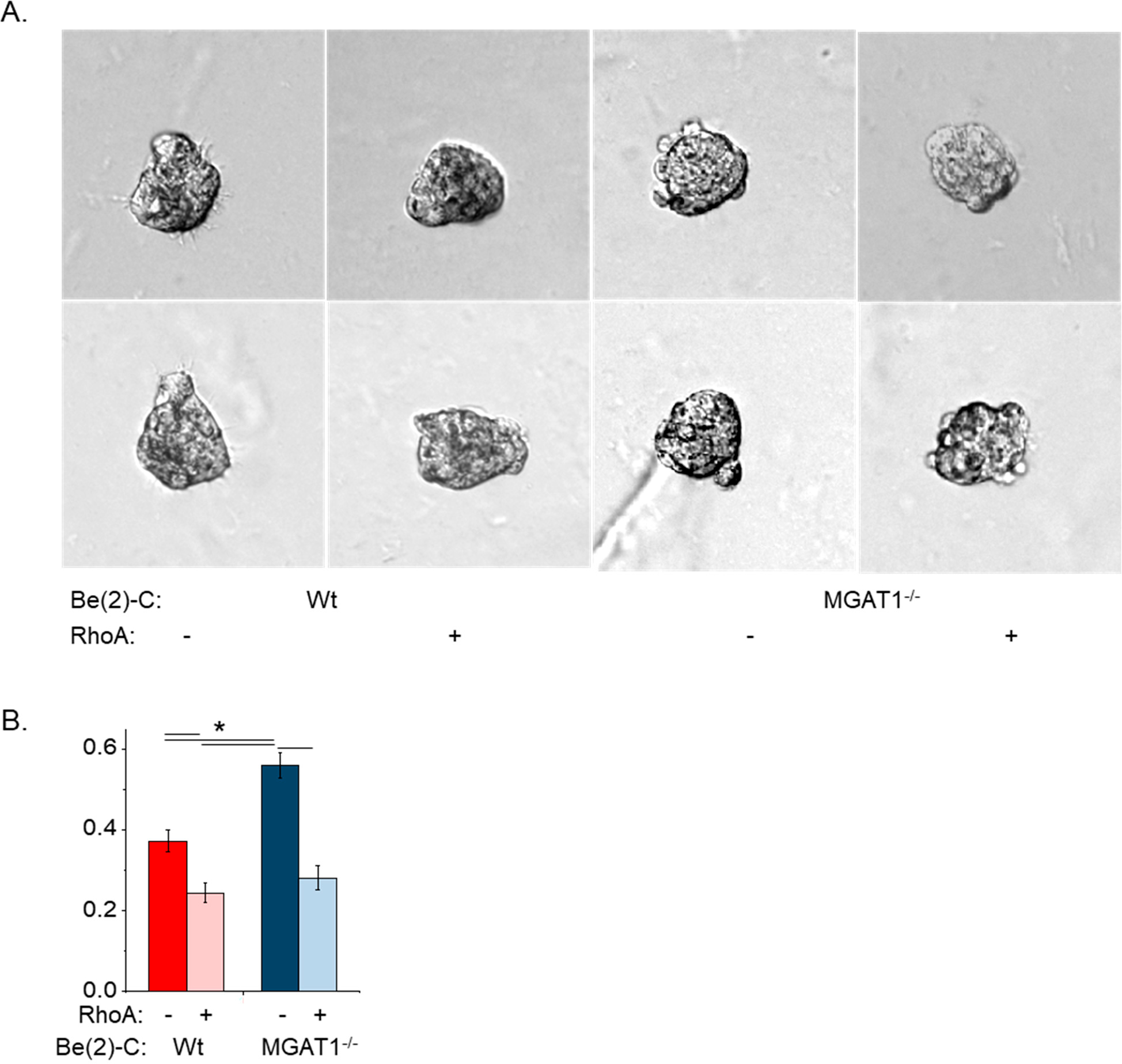
RhoA activation decreased 3D invasion in a glycan-independent manner. (**A**) Micrographs of 3D invading spheroids at 22 h, as indicated. (**B**) Quantification of the invasion area of BE(2)-C without (*n* = 21), BE(2)-C with (*n* = 19), BE(2)-C(MGAT1^−/−^) without (*n* = 22), and BE(2)-C(MGAT1^−/−^) with 1 μg/mL RhoA activator (*n* = 15). *n* denotes the number of invading spheroids. Two-way ANOVA was used with correction; * *p* < 0.05. All quantifications are represented as the mean ± SEM.

Previously, we showed that cell invasiveness was greatly impacted by 2D versus 3D NB cell cultures [8]. After a 22 h incubation of spheroid cells in extracellular matrix, micrographs were obtained of BE(2)-C and BE(2)-C(MGAT1^−/−^) (Figure 7A). Images revealed that *MGAT1* mutant NB cells had more and longer protrusions than parental NB cells. Quantification of the invasiveness revealed that the mutant cell line was close to about 5.8-fold more invasive than the parental cell line (Figure 7B). These results indicate that the glycosylation mutant cells solely expressing oligomannose type N-glycans were more invasive than those expressing hybrid or complex types of N-glycans. Invasion assays using cell spheroids were also conducted in serum-free medium with or without 20 ng/mL EGF. Representative images showed that both parental and mutant cells had decreased invasiveness relative to untreated cells (Figure 7C). The mutant and Wt cell lines were quite similar, and upon addition to EGF, their levels reduced to a similar extent (Figure 7D). Since the cell spheroid protrusions differed between the cell lines, we evaluated the contribution of RhoA, a regulator of actin cytoskeleton rearrangement [40], to cell invasiveness. Typical images reveal that the *MGAT1* mutant cells had increased cell invasiveness compared with untreated cells and that addition of EGF to the cell lines caused reductions in the number of protrusions of the cell spheroids (Figure 8A). Quantification verified that cell invasiveness was reduced to comparable levels for both cell lines upon treatment with the RhoA activator (1 μg/mL), but BE(2)-C(MGAT1^−/−^) cells were more invasive than the parental cell line (Figure 8B). Taken together, the invasive studies using standard 3D cell culturing conditions show that oligomannose N-glycans greatly increase cell invasiveness. Invasiveness was slightly greater in NB cells with oligomannose N-glycans relative those with complex N-glycans using serum-free conditions (BE(2)-C, 0.38 ± 0.02, *n* = 82; BE(2)-C(MGAT1^−/−^), 0.47 ± 0.02, *n* = 83). Moreover, the addition of either EGF or RhoA markedly reduced invasiveness regardless of the N-glycan type.

## 4. Discussion

To further explore the role of oligomannose N-glycans in NB progression, we have engineered a human NB cell line with a functional loss of GnT-I. Although loss of GnT-I (or mutations in *MGAT1*) are not common in NB or cancer, functional loss of GnT-I, here achieved by CRISPR/Cas9 knockout of *MGAT1*, is a highly efficient approach to produce cells or organisms greatly enriched in oligomannose N-glycans. Introduction of frameshift mutations into *MGAT1* resulted in premature stop codons, and thus truncated GnT-I proteins, which caused a significant reduction in GnT-I activity, and markedly increased expression of oligomannose N-glycans, as observed by the lectin blots. Loss of GnT-I prevents the production of hybrid and complex N-glycans, meaning that N-glycosylated proteins, including the EGFR, will be enriched with oligomannose N-glycans. The high levels of oligomannose N-glycans reduced cell proliferation in standard 2D and 3D cell cultures; however, they greatly increased cell invasiveness in 3D cell cultures. These findings were similar to those reported in a rat NB cell line where GnT-I activity was virtually nullified [8], which supports the hypothesis that increased oligomannose N-glycan levels promote NB invasiveness across species. Moreover, assessment of oligomannose N-glycan levels in human (BE(2)-C, human BE(2)-M17), and rat (NB_1) NB cell lines revealed that higher levels of oligomannose produced greater NB cell invasiveness [32]. Additionally, we compared the effect of EGFR signaling on cell proliferation and invasion between Wt and mutant *MGAT1* BE(2)-C cells. We showed that complex N-glycans commonly linked to N-glycosylation sites of EGFR in BE(2)-C were replaced with oligomannose N-glycans in BE(2)-C(MGAT1^−/−^). Although higher levels of oligomannose N-glycans reduced cell proliferation [8,32], we showed that EGF stimulation caused a large increase in cell proliferation in the BE(2)-C(MGAT1^−/−^) cell line, while it lacked an effect on the BE(2)-C cell line. In both NB cell lines, EGF stimulation caused marked decreases in cell invasiveness, like those observed when cells were treated with a RhoA kinase activator. Thus, our current and past studies establish that increased oligomannose N-glycans in NB cell lines correspond to NBs that are highly invasive and capable of EGF-stimulated proliferation.

N-glycosylated proteins often alter their properties and function when changes occur in the N-glycosylation pathway, as N-glycans in proteins have critical biological roles that are vital for organismal survival [41]. EGFR, a GnT-II protein substrate [27,42–44], is highly expressed in NB cells, and it is quite abundant in NB tissues [28,29]. Autophosphorylation of EGFR in response to EGF stimulation is able to initiate cell proliferation by activating numerous cell signaling pathways (i.e., ERK/MAPK, AKT-PI3K, and PLC-γ-PKC) [21]. Currently, N-glycan processing studies of the EGFR in NB are lacking, but studies using chemical inhibition of the N-glycosylation process or more directed changes to the N-glycans by overexpression or knockdown/out of glycosyltransferase in other cell lines have shown that modified N-glycosylation alters EGFR activation and downstream signaling [23,25–27,31]. Further studies that directly addressed oligomannosylated EGFR found decreased localization of heterologous oligomannosylated EGFR to the plasma membrane in CHO cells [24], and another study concluded that reduced amounts of complex N-glycans impacted ganglioside GM3-mediated inhibition of EGFR phosphorylation in human ovarian epidermoid carcinoma A431 cells [31]. In human NB cells, we showed that oligomannosylated EGFR levels under either 2D or 3D cell culture were unaltered, and that EGFR from BE(2)-C(MGAT1^−/−^) were more prone to higher levels of autophosphorylation with or without EGF stimulation, which is likely why BE(2)-C(MGAT1^−/−^) cells were able to undergo EGF-stimulated proliferation while BE(2)-C cells were not. Further 3D cell cultures were much more sensitive to EGF treatment. Thus, our studies provide evidence that substitution of complex N-glycans with oligomannose N-glycans in NB cells promotes NB growth and, furthermore, support the importance of using 3D cell cultures, instead of 2D cell cultures, when evaluating NB growth.

EGFR is a single-pass transmembrane protein with an N-terminal extracellular region and a cytoplasmic region [21,23]. The extracellular region consists of four domains (I–IV), and each domain has up to four N-glycosylation sites, while the cytoplasmic region contains the tyrosine kinase domain and the C-terminal tail with phosphorylated Tyr residues [23]. Domains I and III form the EGF binding site, while domain II and the N-terminal region of the transmembrane (TM) segment are involved in dimerization [21]. Domain IV is attached to the TM and can interact with domain II via disulfide bonds [21]. We speculate that there are subtle changes to the structure of the extracellular domain of EGFR due to the replacement of complex N-glycans with oligomannose N-glycans, as this substitution would decrease the hydrophilicity and increase the dynamics of the extracellular domains, thus altering EGF binding. Moreover, these structural changes in the extracellular region, including those in domain II, could alter the placement of the transmembrane segment in the plasma membrane, which would alter dimerization and, subsequently, could result in subtle structural changes in the cytoplasmic portion of the protein. The scenario supported by our results would be that the 3-dimensional structure of oligomannosylated EGFR would be more inclined to dimerize, have increased autophosphorylation without or with EGF stimulation, and be more prone to undergoing EGF-stimulated proliferation.

In this study, we utilized 3D cell culture conditions, which more closely represent the physiological conditions found in the solid tumors of NB. Traditional monolayered 2D cell cultures have equal exposure to nutrients and oxygen, while cell spheroids are composed of subpopulations—the outer proliferative/invasive layers and the inner necrotic/hypoxic core—which is more representative of solid tumors [45]. 3D cell cultures also better represent the heterogeneity of NB, as previous studies have shown variations in the expression of a tumor-associated antigen in carcinoma [46], protein expression patterns in human NB cell lines [47], and cancerous cellular processes [48]. Here, again, we highlight the importance of considering 3D cell cultures as we show that cell invasiveness was reduced by slightly more than half in human NB cells when complex N-glycans were substituted with oligomannose N-glycans under 2D standard cell culturing conditions; however, there was a great increase (about 5.8-fold) in cell invasiveness under 3D standard cell culturing conditions. Previously, we made this same observation in rat NB cells and therefore proposed that 3D cell cultures are more likely to mimic NB tumors [8]. Even under serum-free conditions of 3D cell cultures, we observed that BE(2)-C(MGAT1^−/−^) cell invasiveness was greater (at least 1.2-fold) than that by BE(2)-C. Our finding that decreased complex type N-glycans promote cell invasiveness under 3D cell culture also agree with the clinical analysis of NB tumors in patients, which reported that decreased expression of *MGAT5* is linked to more aggressive NB [49]. Therefore, these results indicate that cell invasiveness depends on N-glycan populations and, furthermore, establish that oligomannose N-glycans promote NB cell invasiveness. By utilizing models that better represent physiological conditions found in NB patients, our findings become more translationally relevant and accurate; here, we accomplish this by utilizing 3D cell spheroids.

Although cell invasiveness was N-glycan dependent, we found significant declines in cell invasiveness upon EGF or RhoA activator treatment that was unconnected to N-glycan types. It was shown that when both cell lines were treated with either EGF or RhoA, their invasiveness decreased to similar levels. Previous reports have shown that EGFR activation mediates actin dynamics via RhoA signaling in various cancers [50–52]. Hence, our results argue that EGF-activated EGFR contributes to decreased cell invasiveness in a mechanism involving RhoA activation in an N-glycan independent manner.

Currently, the involvement of RhoA signaling in NB dissemination is contradictory. ROCK2 levels have been shown to be higher in patients with a poor NB prognosis [53]. Moreover, the authors suggested that this was due to RhoA activation; however, activated RhoA was not directly measured to show that it is higher in the patients with a poor prognosis. Since studies have suggested that the ROCK proteins have opposing roles in the maturation of focal adhesion complexes and stress fiber formations [54–56], it is unclear whether activated RhoA was higher in these patients. RhoA signaling in NB cell invasion has also been studied. One study using dispersed human SK-N-Be(2) NB cells showed that RhoA activation via neuropeptide Y/Y5 receptor increased cell invasion [57], while another study showed that increased RhoA activation caused a decline in NB cell invasion using human SK-N-Be(2) NB spheroids [58]. The discrepancy is likely due to the earlier study using 2D cell culture instead of 3D cell culture. Thus, our study agrees with the latter study that RhoA activation suppresses cell invasiveness, and that RhoA activation is independent of N-glycan types.

## 5. Conclusions

Herein, we augment our prior studies that demonstrated that oligomannose N-glycans promote cell invasiveness in rat NB cells [8] to include a human NB cell line. Additionally, we have proven that NB cells with high levels of oligomannose N-glycans are sensitized to EGF-stimulated cell proliferation. In both cases, our research emphasizes the importance of using 3D cell culturing conditions when measuring cancerous properties, such as cell proliferation and invasion. Taken together, we corroborate that enrichment of oligomannose type of N-glycans contributes to NB development and progression.

## Supplementary Material

supplementary material

## Data Availability

Data are available upon request.
