# Supplementary material for: Reduction of N-Acetylglucosaminyltransferase-I Activity Promotes Neuroblastoma Invasiveness and EGF-Stimulated Proliferation In Vitro
Source: Int J Transl Med (Basel). Author manuscript; Available in PMC 2024 Dec 31. (PMC11687401; doi:10.3390/ijtm4030035)
Supplement: supplementary material [file NIHMS2040161-supplement-supplementary_material.zip › supplementary material 240611.pptx]

## Slide 1
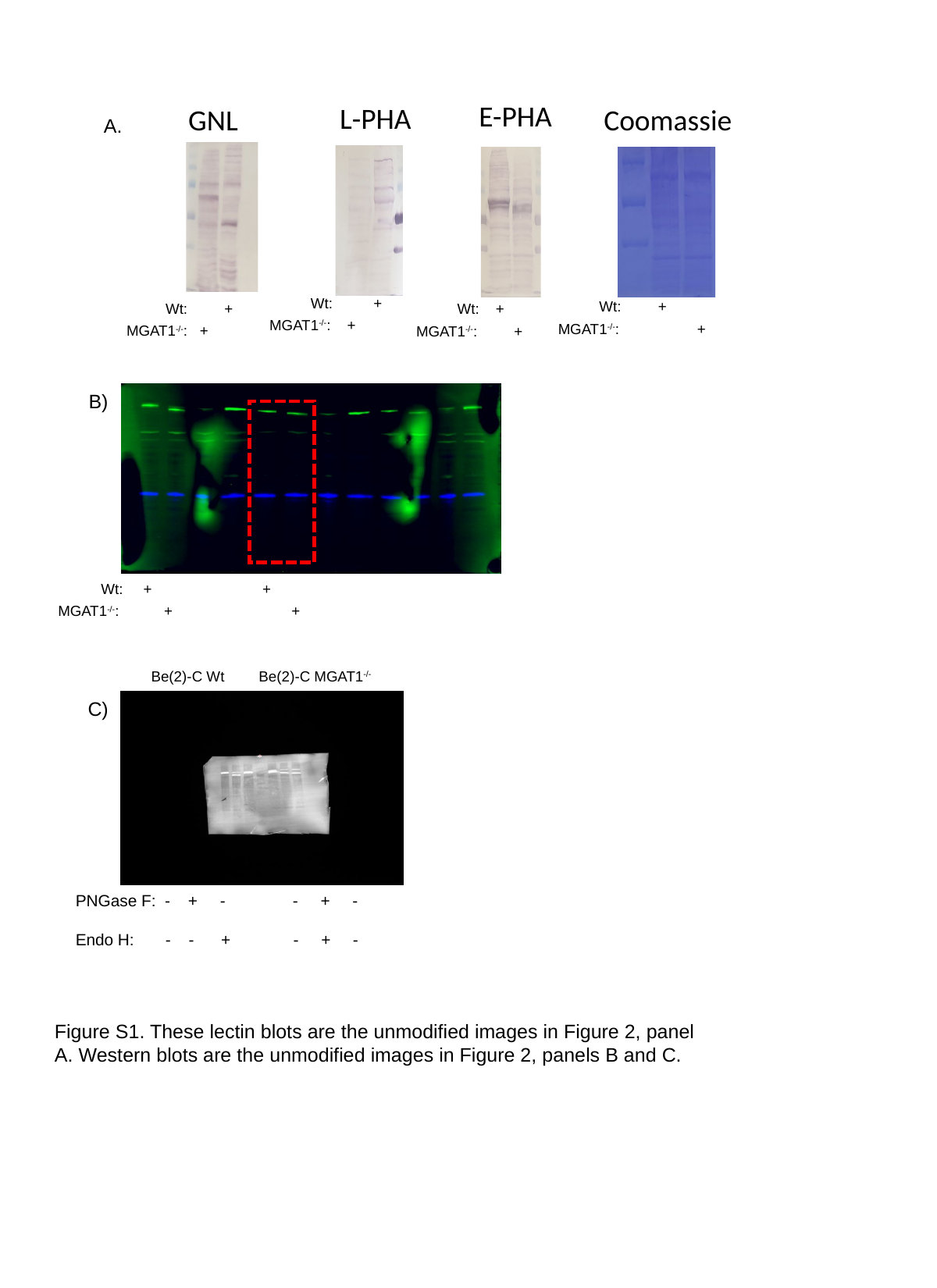

E-PHA
L-PHA
GNL
Coomassie
A.
Wt: +
MGAT1-/-: +
Wt: +
MGAT1-/-: +
Wt: +
MGAT1-/-: +
Wt: +
MGAT1-/-: +
B)
Wt: + +
MGAT1-/-: + +
Be(2)-C Wt
Be(2)-C MGAT1-/-
C)
PNGase F: - + - - + -
Endo H: - - + - + -
Figure S1. These lectin blots are the unmodified images in Figure 2, panel A. Western blots are the unmodified images in Figure 2, panels B and C.

## Slide 2
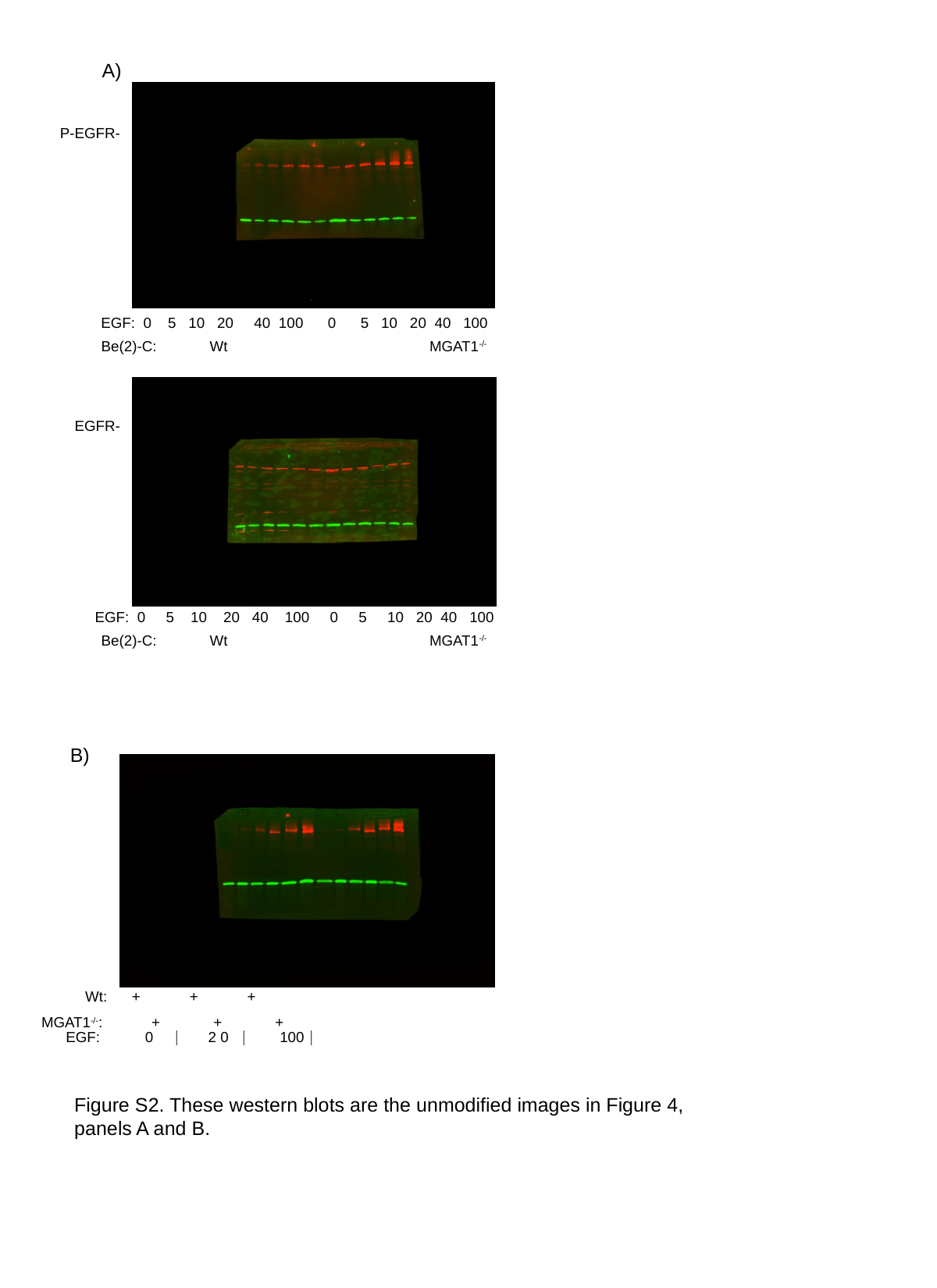

A)
Unmodified images of Figure 4
P-EGFR-
EGF: 0 5 10 20 40 100 0 5 10 20 40 100
Be(2)-C: Wt 	 MGAT1-/-
EGFR-
EGF: 0 5 10 20 40 100 0 5 10 20 40 100
Be(2)-C: Wt 	 MGAT1-/-
B)
Wt: + + +
MGAT1-/-: + + +
EGF: 0  2 0  100 
Figure S2. These western blots are the unmodified images in Figure 4, panels A and B.

## Slide 3
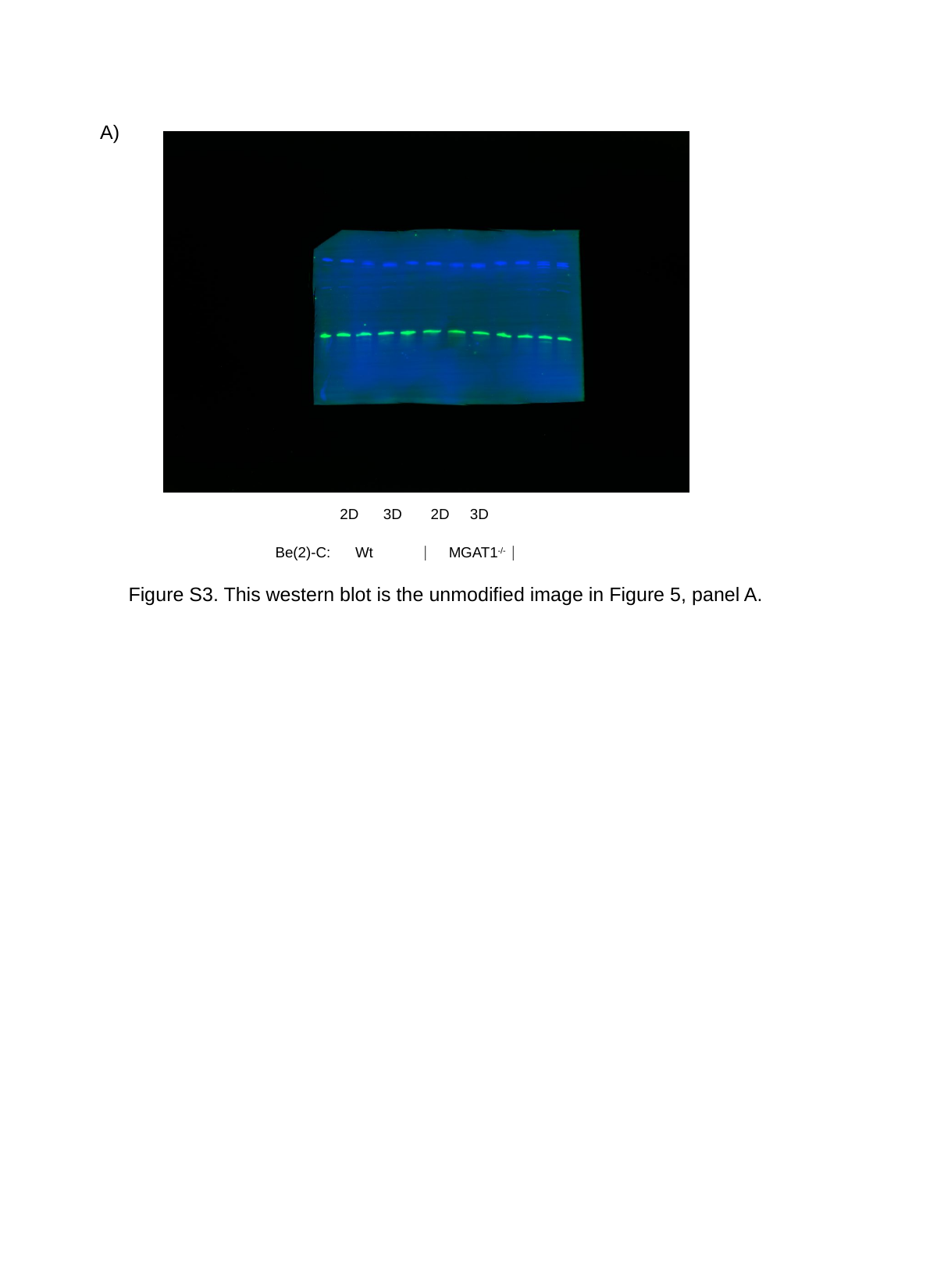

A)
2D 3D 2D 3D
Be(2)-C: Wt  MGAT1-/- 
Figure S3. This western blot is the unmodified image in Figure 5, panel A.
